# BDA‐410 inhibits SARS‐CoV‐2 main protease activity and viral replication in mammalian cells

**DOI:** 10.1111/jcmm.17442

**Published:** 2022-09-09

**Authors:** Christopher Schwake, Lindsay McKay, Anthony Griffiths, Christina Scartelli, Robert Flaumenhaft, Athar H. Chishti

**Affiliations:** ^1^ Department of Developmental, Molecular, and Chemical Biology, Graduate School of Biomedical Sciences Tufts University School of Medicine Boston Massachusetts USA; ^2^ National Emerging Infectious Diseases Laboratories (NEIDL), Department of Microbiology Boston University Boston Massachusetts USA; ^3^ Division of Hemostasis and Thrombosis, Department of Medicine, Beth Israel Deaconess Medical Center Harvard Medical School Boston Massachusetts USA

**Keywords:** BDA‐410, Calpain‐1, COVID‐19, SARS‐CoV‐2

Severe acute respiratory syndrome coronavirus 2 (SARS‐CoV‐2) is a novel highly virulent coronavirus that causes severe illness and mortality in certain patients. SARS‐CoV‐2 contains a positive‐sense single‐stranded RNA genome that encodes two essential cysteine proteases, a papain‐like (PLpro) and 3C‐like main protease (3CLpro). These proteases cleave the nonstructural polyproteins essential for viral maturation and replication.^1^, ^2^ Recently, much interest has been focused on identifying novel protease inhibitors against SARS‐CoV‐2.^3^, ^4^, ^5^, ^6^, ^7^ The essentiality of SARS‐CoV‐2 viral cysteine proteases prompted us to test a pharmacological inhibitor of the cysteine protease calpain‐1 against recombinant 3CLpro and SARS‐CoV‐2 infection in mammalian cells.

There is a precedent in using calpain inhibitors II and XII against SARS‐CoV‐2 3CLpro.^8^ BDA‐410 (Mitsubishi Pharma) is a cysteine protease inhibitor that was originally developed as a relatively selective inhibitor of calpain‐1 cysteine protease activity. It has been examined as a therapeutic intervention in neuronal cell death using Alzheimer's disease models^9^, ^10^ and as an anti‐malarial drug.^11^ BDA‐410 is an orally active, non‐toxic, non‐carcinogenic and non‐teratogenic chemical inhibitor that has been evaluated for dose and toxicity parameters in rat, dog and monkey models. Here, we provide the first evidence that BDA‐410 inhibits SARS‐CoV‐2 main protease activity and attenuates live viral replication in the VeroE6 mammalian cell line.

BDA‐410 with the chemical name (2S)‐N‐{(1)‐l‐[(S)‐hydroxy(3‐oxo‐2‐phenyl‐1‐cyclopropen‐1‐yl) methyl]‐2‐methylpropyl}‐2‐benzenesulfony‐lamino‐4‐methylpentanamide (C26H32N2O5S) is a synthetic cysteine protease inhibitor (MW 484.6 Da). BDA‐410 selectively inhibits calpain‐1 (Ki = 130 nM) compared to calpain‐2 (Ki = 630 nM). The IC50 values for papain (400 nM), cathepsin B (16 μM), thrombin (100 μM), cathepsin D (91.2 μM), cathepsin G (100 μM) and proteasome 20S (100 μM) inhibition indicate the utility of BDA‐410 as a potentially broad inhibitor of cysteine proteases at higher concentrations. The toxicology and mutagenicity studies of BDA‐410 are favourable for use in therapeutic applications.^11^ We performed molecular docking simulation to assess the efficacy of BDA‐410 against viral cysteine protease. The chemical structure of BDA‐410 was converted to SMILES format and then to PDB format before docking with SARS‐CoV‐2 3CLpro (PDB: 6YB7) using AutoDock Vina within Chimera with minimized structures. Molecular docking of BDA‐410 (Figure 1A) in the active site of the SARS‐CoV‐2 3CLpro (PDB: 6YB7) showed BDA‐410 forming three potential binding poses (Figure 1B). The binding pose with the highest score (−6.9) showed stable pi‐stacking interactions with the two benzene rings (Figure 1C). Of interest, there are two predicted stabilizing hydrogen bonds between BDA‐410 and the catalytic residue CYS145 due to proximity of hydrogen bonding functional groups (Figure 1C).

**FIGURE 1:**
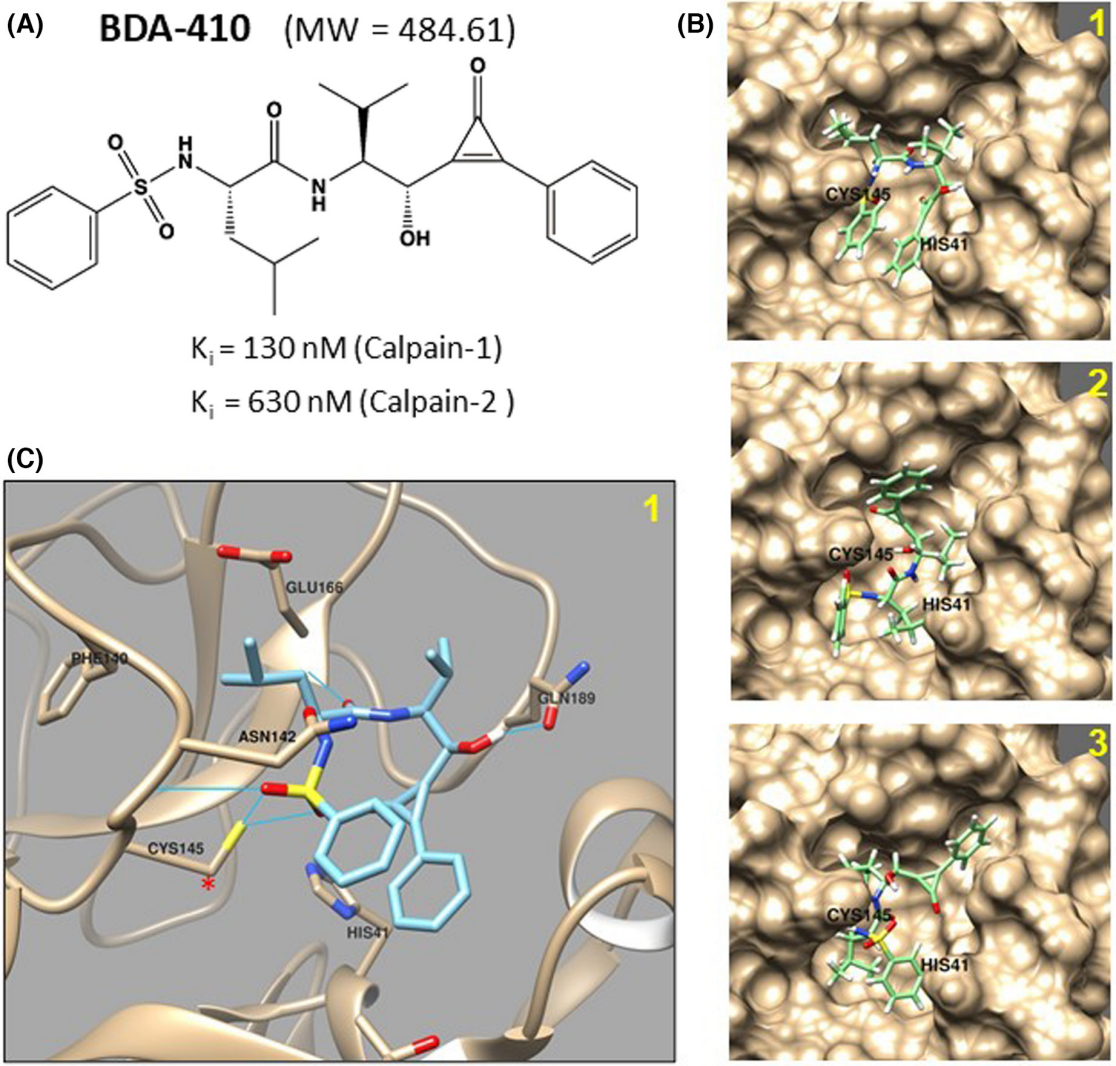
Molecular docking of BDA‐410 to SARS‐CoV‐2 main protease. (A) Chemical structure of the cysteine protease inhibitor, BDA‐410. (B) Molecular docking simulation of BDA‐410 with the active site of SARS‐CoV‐2 3CLpro (PDB: 6YB7). The three best binding poses are shown. (C) Active site from pose 1 in (B). Blue lines represent hydrogen bonds between BDA‐410 and active site residues. The catalytic cysteine (CYS145) is denoted by the red asterisk

Recognizing that SARS‐CoV‐2 encodes two essential cysteine protease enzymes required for its replication in mammalian cells, we first tested the efficacy of the cysteine protease inhibitor, BDA‐410, on the SARS‐CoV‐2 main protease 3CLpro using a fluorescence‐based assay. A Förster resonance energy transfer (FRET)‐based assay was used to measure recombinant 3CLpro enzyme activity by using 5 mM of the dual‐labelled substrate, DABCYL‐KTSAVLQ↓SGFRKM‐EDANS (GL Biochem, Shanghai), which contains a protease specific cleavage site after the glutamine residue (*Q*). Our results showed that BDA‐410 inhibited 3CLpro activity in a dose‐dependent manner (Figure 2A).

**FIGURE 2:**
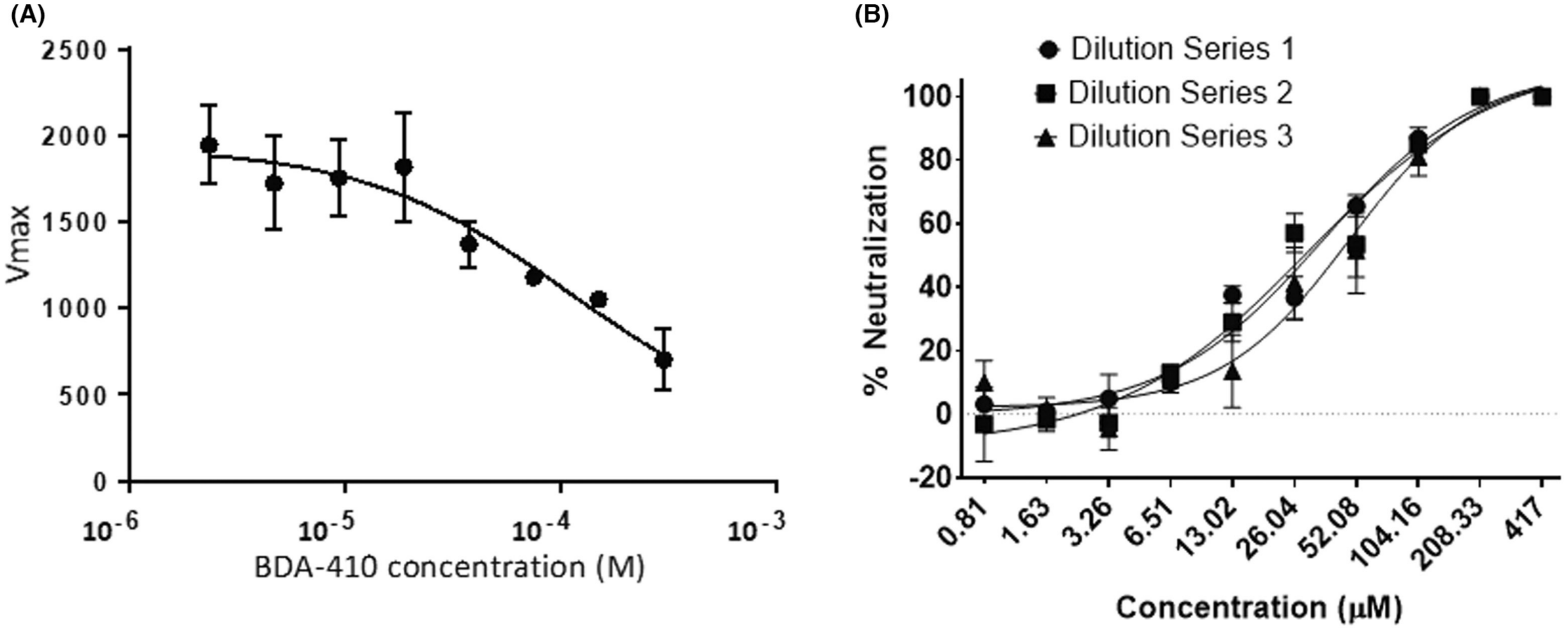
Effect of calpain‐1 inhibitor BDA‐410 on SARS‐CoV‐2 main protease and viral replication. (A) Fluorescence based 3CLpro enzymatic activity assay with BDA‐410. In the intact peptide, EDANS fluorescence is quenched by the DABCYL group. Following enzymatic cleavage by 200 nM SARS‐CoV‐2 3CLpro, a fluorescent product is produced and monitored (Ex/Em = 340/460 nm). (B) Viral neutralization assay. A viral neutralization assay determined the efficacy of BDA‐410 against SARS‐CoV‐2 in the NR‐596 VeroE6 cell line. After staining with 0.2% crystal violet, the plaques were counted and recorded, and the neutralization potency of BDA‐410 was quantified. All viral infection quantification assays were performed under BSL‐4 at the National Emerging Infectious Diseases Laboratories (NEIDL)

Next, we used a viral neutralization assay to determine the efficacy of BDA‐410 against SARS‐CoV‐2 in the NR‐596 VeroE6 cell line. To quantify plaques, an Avicel plaque reduction assay was employed. In brief, BDA‐410 (suspended in DMSO) was diluted in DPBS from 5000 to 1667 μM. Dilutions were incubated in a 5% CO_2_ incubator at 37°C for 1 h with 1000 plaque‐forming units/ml (PFU/ml) of SARS‐CoV‐2 (isolate USA‐WA1/2020). The final starting dilution of the inhibitor was 417 μM and serially diluted twofold. The amount of DMSO was equalized between dilutions, and dilutions of each sample were prepared in triplicate, and then, each dilution was plated in triplicate (DMSO <0.1%). Two negative controls of DPBS or DPBS mixed with DMSO and two positive controls of 1000 PFU/ml SARS‐CoV‐2 incubated with DPBS or 1000 PFU/ml SARS‐CoV‐2 incubated with DPBS mixed with DMSO were included. For controls containing DMSO, the DMSO was diluted to the same degree as the inhibitor (e.g., diluted 1/3 in DPBS) before mixing. Dilution or control samples were added to confluent monolayers of NR‐596 Vero E6 cells and incubated in a 5% CO_2_ incubator at 37°C for 1 h. After incubation for 1 h, the inoculum was removed, and 2 ml was added to each well with incubation for 48 h at 37°C/5% CO_2_. The fixed plates were stained with 0.2% aqueous Gentian Violet in 10% neutral buffered formalin before plaque counting. Quantification of viral infection in vitro was performed in the biosafety level 4 (BSL‐4) facility at the NEIDL in Boston University, MA. The complete SARS‐CoV‐2 neutralization was observed at 208 μM, and the IC_50_ of the BDA‐410 inhibitor ranges from 30.4 to 48.2 μM (Figure 2B). This is the first demonstration of BDA‐410 as a potential inhibitor of SARS‐CoV‐2 replication. BDA‐410 inhibited SARS‐CoV‐2 main protease and viral replication in a neutralization assay. BDA‐410 has an attractive safety profile and its inhibition of SARS‐CoV‐2 main protease activity and viral replication make it a potential lead candidate for preclinical drug development.

Viral entry of both MERS‐CoV and SARS‐CoV has been shown to utilize host cell cysteine proteases cathepsin B and L during endosomal uptake.^12^ Recent studies identified SARS‐CoV‐2 as dependent on cathepsin L for entry.^13^ While BDA‐410 shows high potency against calpain‐1 (Ki = 130 nM), it can also inhibit cathepsins at micromolar concentrations.^10^ Therefore, broad inhibition of cysteine protease activity by BDA‐410 may encompass additional therapeutic effects by suppressing host cathepsins along with SARS‐CoV‐2 protease activity by exerting a dual effect targeting both the essential viral cysteine proteases and required host cellular endosomal uptake machinery. While we are aware that BDA‐410 completely neutralized SARS‐CoV‐2 at relatively high inhibitor concentrations, these measurements could be rationalized in the context of disease treatment such as in sickle cell disease (SCD). Our serendipitous interest in testing BDA‐410 as an inhibitor of SARS‐CoV‐2 originates from previous demonstration of its efficacy against malaria^11^ and SCD.^14^ Recent FDA approval of Voxelotor, a modulator of haemoglobin oxygen‐affinity, for the treatment of SCD is recommended for administration at 900–1500 mg daily and indefinitely.^15^ Treatment for COVID‐19 would be acute, and thus permitting a higher dose of BDA‐410 (~18 mg daily) over a relatively short period consistent with its toxicity profile. This dose was estimated from the IC50 value shown in Figure 2B. The unprecedented speed and efficacy of the vaccines developed against COVID‐19 and its variants suggest that development of small molecule inhibitors against severe COVID‐19 will be an essential tool in the continued fight against this deadly disease.

Pharmacological inhibition of cysteine protease activity holds great promise as a potential drug intervention against severe COVID‐19 due to the essentiality of the two cysteine proteases encoded in the SARS‐CoV‐2. The recent demonstration of SARS‐CoV‐2 main protease inhibition by nirmatrelvir,^16^ and its combination with ritonavir (PAXLOVID™),^17^ provides compelling evidence for the treatment of COVID‐19. BDA‐410 with an established safety profile has been previously examined as a potential inhibitor for several disease indications. Its relative specificity against calpain‐1 is consistent with the inhibition of purified SARS‐CoV‐2 main protease activity and attenuation of live virus in a neutralization assay. Our findings suggest that the development of BDA‐410 derivatives and their in vivo evaluation in relevant animal models will likely lay the foundation for novel treatments against severe COVID‐19 in patients afflicted with and without lesions such as sickle cell disease and malaria.

## AUTHOR CONTRIBUTIONS

**Athar Chishti:** Conceptualization (equal); funding acquisition (equal); supervision (equal); writing – review and editing (equal). **Christopher Schwake:** Conceptualization (equal); methodology (equal); writing – original draft (equal); writing – review and editing (equal). **Lindsay McKay:** Data curation (equal); methodology (equal). **Anthony Griffiths:** Methodology (equal); project administration (equal); writing – review and editing (equal). **Christina Scartelli:** Data curation (equal); methodology (equal). **Robert Flaumenhaft:** Methodology (equal); writing – review and editing (equal).

## CONFLICT OF INTEREST

The authors declare no competing financial interests.

## Data Availability

Data sharing not applicable ‐ no new data generated
